# Responses of community-level plant-insect interactions to climate warming in a meadow steppe

**DOI:** 10.1038/srep18654

**Published:** 2015-12-21

**Authors:** Hui Zhu, Xuehui Zou, Deli Wang, Shiqiang Wan, Ling Wang, Jixun Guo

**Affiliations:** 1 Key Laboratory of Vegetation Ecology, Ministry of Education, Institute of Grassland Science, Northeast Normal University, Changchun, Jilin 130024, China; 2 School of Life Sciences, Northeast Normal University, Changchun, Jilin 130024, China; 3 Jilin Provincial Key Laboratory of Animal Resource Conservation and Utilization, Northeast Normal University, Changchun, Jilin 130024, China; 4 State Key Laboratory of Cotton Biology, College of Life Sciences, Henan University, Kaifeng, Henan 475004, China

## Abstract

Climate warming may disrupt trophic interactions, consequently influencing ecosystem functioning. Most studies have concentrated on the temperature-effects on plant-insect interactions at individual and population levels, with a particular emphasis on changes in phenology and distribution. Nevertheless, the available evidence from the community level is limited. A 3-year field manipulative experiment was performed to test potential responses of plant and insect communities, and plant-insect interactions, to elevated temperature in a meadow steppe. Warming increased the biomass of plant community and forbs, and decreased grass biomass, indicating a shift from grass-dominant to grass-forb mixed plant community. Reduced abundance of the insect community under warming, particularly the herbivorous insects, was attributed to lower abundance of *Euchorthippus unicolor* and a Cicadellidae species resulting from lower food availability and higher defensive herbivory. Lower herbivore abundance caused lower predator species richness because of reduced prey resources and contributed to an overall decrease in insect species richness. Interestingly, warming enhanced the positive relationship between insect and plant species richness, implying that the strength of the plant-insect interactions was altered by warming. Our results suggest that alterations to plant-insect interactions at a community level under climate warming in grasslands may be more important and complex than previously thought.

Plants and insects are extremely diverse, and plant-insect interactions are among the pivotal ecological and evolutionary trophic interactions in terrestrial ecosystems^1^,^2^. Plant-insect interactions are immensely complex, with multiple species interacting over a range of trophic levels through predation, parasitism, and pollination^3^,^4^,^5^. These interactions can profoundly affect community structure and ecological functioning^6^,^7^, and regulate the responses of ecosystems to climate change^8^. Unfortunately, plants and insects are increasingly affected by climate warming, which alters their interactions^9^. Numerous studies have revealed that phenological shifts at the individual and species levels caused by elevated air temperature markedly alter the trophic interactions between plants and insects^10^,^11^,^12^, and disrupt widespread ecological communities. There is increasing concern that the responses of plants and insects to climate warming may be modulated within a community context^13^, which would likely further influence their interactions. However, an empirical understanding of the temperature effects on plant-insect interactions at the community scale remains rudimentary^14^.

In the context of climate warming, the responses of plant and insect communities have been considered separately. A growing body of evidence from empirical studies has documented that elevated temperature has the potential to alter plant communities, in terms of diversity and composition^15^,^16^,^17^. However, little direct evidence is available to show that climate warming influences insect communities^18^,^19^, and most information on temperature-mediated effects on the diversity and abundance of insects is derived from studies on how insect assemblages are affected along altitudinal and latitudinal gradients (proxies for temperature influence)^20^,^21^,^22^. Recent experiments have shown that climate-mediated changes in plants or insects are not isolated, but rather connected at the same, adjacent, and even higher trophic levels^9^, because plant and insect communities are closely linked^23^,^24^. For example, plant communities with higher species richness and/or productivity provide a favorable quality and/or greater quantity of resources for insects, thus increasing their species richness and abundance^26^,^25^. Similarly, structural heterogeneity in diverse and productive plant communities is found to affect insect communities as well^27^,^28^. Nevertheless, whether and how alterations to plant communities under climate warming may cascade to influence insect communities remains poorly understood, thus posing major challenges for understanding plant-insect associations at the community level under climate warming scenarios.

Herein we examine the effects of climate warming on plant and insect communities, and interactions, using a field experiment with manipulated air temperatures to test hypotheses specific to various mechanistic pathways (Fig. 1). The studied meadow steppe is located in northeastern China in the eastern region of the Eurasian grassland biome, and it has experienced dramatic increases in temperature over recent decades^29^. Generally, elevated temperatures will stimulate the primary productivity of plant communities by directly enhancing photosynthesis^30^, thus leading to increase canopy coverage and reduced light availability^31^. However, warming may cause a decline in plant species richness, and rapid losses of plant species richness under climate warming have been widely observed^15^,^32^. Furthermore, warming can change plant community composition^33^, with shifts toward the declining dominance of competitive dominant grasses^16^. Such altered plant community characteristics may cascade to higher trophic levels. Decreased plant species richness may lead to reduced insect species richness, and increased plant biomass may cause enhanced insect abundance. Thus, altered plant and insect communities may influence their interactions within a community context under warming.

## Results

### Vegetation variables

**Figure 2:**
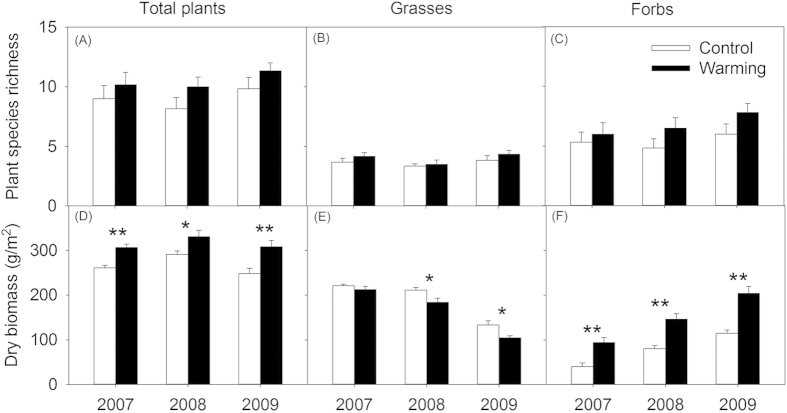
Effects of warming on plant species richness ((**A**) total plants, (**B**) grasses, (**C**) forbs) and biomass ((**D**) total plants, (**E**) grasses, (**F**) forbs) in three experimental years (2007–2009). Values represent means ± standard errors. Asterisk indicates a significant difference between warmed and control treatments in each given year, and ^*^P < 0.05, ^**^P < 0.001.

Interannual changes were not observed in plant species richness, grass species richness, or forb species richness (all *P* > 0.05, Table 1). Experimental warming marginally enhanced the plant species richness by 16.7% and the forb species richness by 25.8% (both *P* < 0.06), but did not affect the grass species richness (Fig. 2A–C, *P* > 0.10). The warming effects on the species richness of the entire plant community, grasses, and forbs did not change with year (all *P* > 0.10).

**Figure 3:**
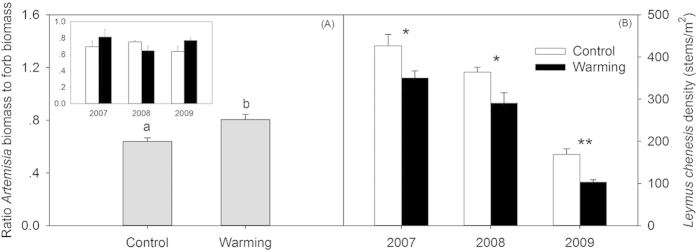
Effects of warming on the density of *Leymus chinensis* and *Artemisia* biomass in three experimental years (2007–2009). Gray columns indicate the mean values for the three years in each treatment, and different lowercase letters indicate significant differences in the control and warming plots. Values represent means ± standard errors. An asterisk indicates a significant difference between warmed and control treatments in each given year, and ^*^P < 0.05, ^**^P < 0.001. Insets represent the responses of the *Artemisis* to forb biomass ratio to elevated temperature in each experimental year.

Strong interannual variations were observed in the aboveground biomass (AGB) of the entire community, grasses, forbs, and *Artemisia* (all *P* < 0.05, Table 1). The community AGB varied from 283.8 g/m^2^ in 2007, to 310.9 g/m^2^ in 2008 and 278.4 g/m^2^ in 2009, and grass AGB changed from 216.7 g/m^2^ in 2007, to 197.8 g/m^2^ in 2008 and 118.9 g/m^2^ in 2009 (Fig. 2D,E). The forb AGB in 2009 (159.5 g/m^2^) was 40.9% and 137.5% greater than that observed in 2007 and 2008, respectively (Fig. 2F), and the *Artemisia* AGB in 2009 (106.1 g/m^2^) was 25.3% and 225.1% higher than that observed in 2007 and 2008, respectively (Fig. 3A). Across the 3 years, experimental warming significantly increased the AGB of the community, forb, and *Artemisia* by 18.0%, 88.9%, and 64.4% (all *P* < 0.001, Table 1), respectively, but decreased the grass AGB by 11.5% (*P* < 0.05). Interactions of warming × year were observed in the AGB of the community, grasses, forbs, and *Artemisia* (all *P* > 0.10).

The interannual alterations in the density of *Leymus chinensis*, a dominant plant species, were significant across the 3 years (*P* < 0.0001). The density of *L. chinensis* decreased annually from 2007 to 2009 (388 stems/m^2^ in 2007, 327 stems/m^2^ in 2008, and 136 stems/m^2^ in 2009), and was significantly lower in the warmed plots (247 stems/m^2^) compared with control plots (319 stems/m^2^). Interactive effects between the year and on the density of *L. chinensis* were not observed (Fig. 3B).

### Insect species richness and abundance

**Figure 4:**
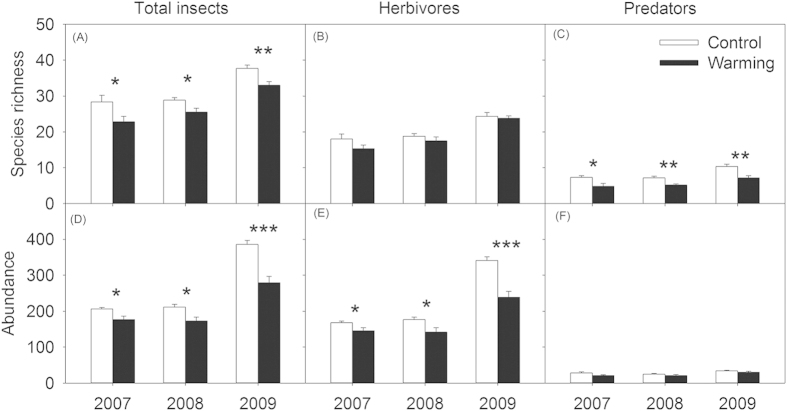
Effects of warming on insect species richness and abundance ((**A**,**D**) total insects, (**B**,**E**) herbivores, (**C**,**F**) predators) in three experimental years (2007–2009). Values represent the means ± standard errors. Asterisk indicates a significant difference between the warmed and unwarmed treatments in each given year, and ^*^P < 0.05, ^**^P < 0.001, ^***^P < 0.0001.

The insect community variables fluctuated dramatically across the 3 years of the experiment. The species richness (total insects, *P* < 0.0001; herbivores, *P* < 0.0001; and predators, *P* < 0.0001) and abundance (total insects, *P* < 0.0001; and herbivores, *P* < 0.0001) increased gradually from 2007 to 2009 (Fig. 4A–E). The abundance of predators in 2009 was higher than that in 2007 and 2008 (*P* < 0.001, Fig. 4F). Experimental warming significantly decreased insect species richness and predator species richness, and the abundance of total insects, herbivores, and predators, compared with that of the controls (Table 1, Fig. 4). In addition, the warming effects changed significantly with the year for the total insect abundance and herbivore abundance (both *P* < 0.001, Fig. 4D,E), but not for the other variables for the total insects, herbivores, and predators (all *P* > 0.10).

**Figure 5:**
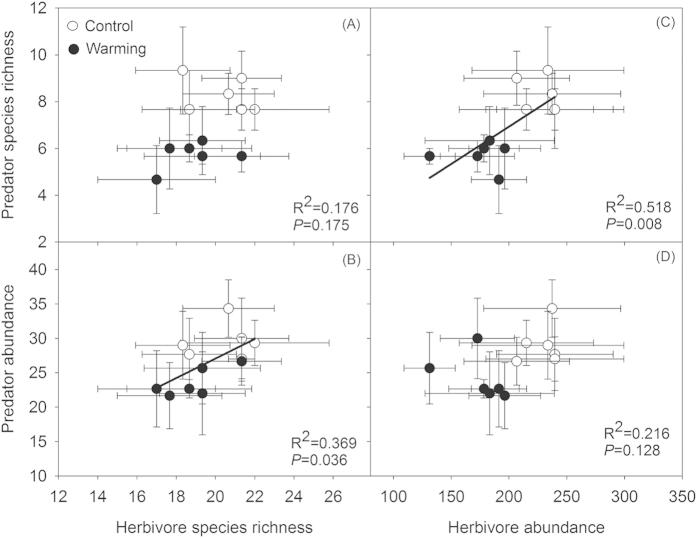
Relationships between herbivores and predators in three experimental years (2007–2009) in all of the treatments. Points are averaged values of variables from three years, resulting in n = 6 in each treatment for further analysis. Values are means ± standard errors. All of the data points were used in simple linear regressions analysis.

The species richness of predators was not related to the species richness of herbivores (Fig. 5A). However, the predator abundance gradually increased with increass in herbivore species richness gradient (Fig. 5B), and the predator species richness was significantly enhanced with increasing herbivore abundance (Fig. 5C). The predator abundance was not closely link to the herbivore abundance (Fig. 5D).

**Figure 6:**
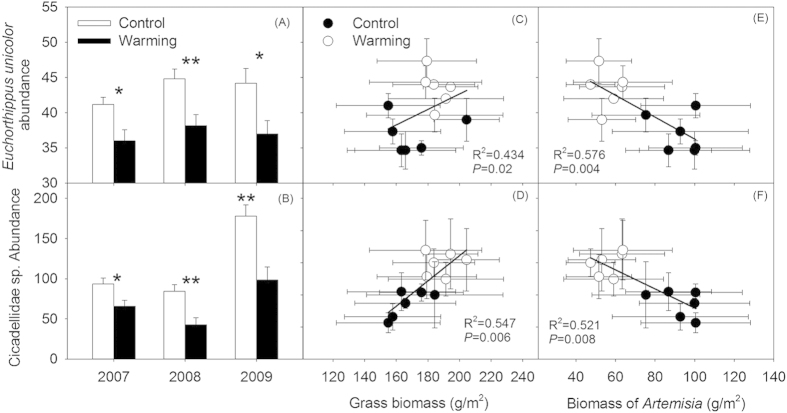
Effects of warming on the abundance of *Euchorthippus unicolor* (**A**) and Cicadellidae sp. (**B**) in three experimental years (2007-2009), and the relationships between their abundance and grass biomass (**C**,**D**) and *Artemisia* biomass (**E**,**F**). Asterisk indicates a significant difference between warmed and unwarmed treatments in each given year, and ^*^P < 0.05, ^**^P < 0.001. Points are averaged values of variables of three years, resulting in n = 6 in each treatment for further analysis. All of the data points were used in simple linear regressions analysis after ln-transformation. Values are means ± standard errors.

The interannual abundance of *Euchorthippus unicolor* did not change (*P* > 0.10, Fig. 6A), although the abundance of Cicadellidae sp. in 2009 was higher than in 2007 and 2008 (*P* < 0.001, Fig. 6B). Experimental warming significantly decreased the abundance of *E. unicolor* and Cicadellidae sp., compared with that of the controls (Table 1). In addition, the warming effects did not change significantly with the year for the abundance of *E. unicolor* and Cicadellidae sp. (all *P* > 0.10).

### Insects related to plant species richness and biomass

**Figure 7:**
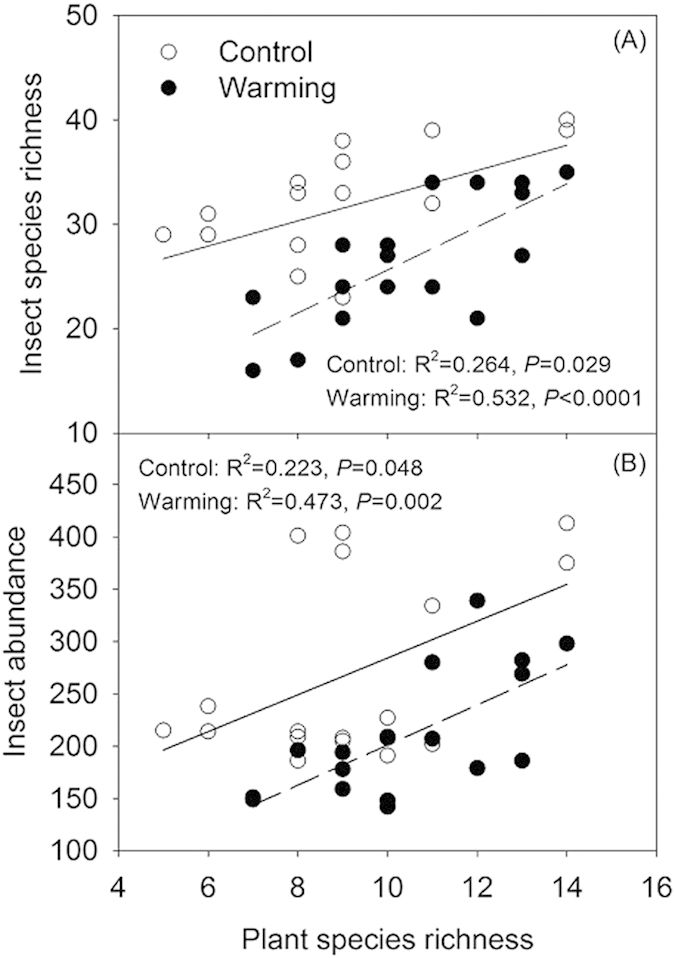
Dependence of insect species richness (**A**) and abundance (**B**) on plant species richness from 2007 to 2009 in the control and warmed treatments. For each treatment, six points were obtained from the replicated plots in each year, resulting in n = 18 in three years for analysis. The data on the insect species richness and abundance were used in the analysis after ln-transformation. Solid lines indicate simple regressions of all data points in the control plots, and dash lines indicated simple linear regressions of all data points in the warmed plots.

*E. unicolor* and Cicadellidae sp. were closely related to plant biomass. The abundance of *E. unicolor* and Cicadellidae sp. markedly increased with the gradient of increasing grass biomass (Fig. 6C,D), but significantly decreased with increase in *Artemisia* biomass gradient (Fig. 6 E,F). The insect species richness linearly increased with increasing plant species richness under both the warming and control treatments (Fig. 7A). Insect abundance was positively dependent on plant species richness in both the warmed and control plots (Fig. 7B). Furthermore, an analysis of covariance (ANCOVA) showed that warming led to a significant increase in the slopes of the linear relationships between insect species richness (*P* < 0.05, Fig. 7A) and abundance (*P* < 0.05, Fig. 7B) when the plant species richness was compared between the treatment and the control.

## Discussion

In the present study, strong interannual variations were observed in certain measured variables in the meadow steppe over the three-year experimental periods (Table 1). The yearly fluctuations of plant AGB and insect species richness and abundance have been reported for other grasslands^16^,^34^. In semiarid regions, water availability limits growth and reproduction of living organisms^35^,^36^. Hence, the interannual variability of the measured variables could have been largely ascribed to yearly changes in precipitation. The total growing season precipitation in 2008 (382.9 mm) was greater than that in 2007 (207.9 mm) and 2009 (286.2 mm), which resulted in the highest plant community biomass in 2008 (Fig. 2D). However, our results showed that other measured variables, such as grass biomass and forb biomass did not present the same fluctuations with plant AGB (Fig. 2E,F), with grass biomass gradually decreasing and forb biomass gradually increasing from 2007 to 2009. Interannual variations in the biomass of grasses and forbs could have been mainly caused by the competition among plant species induced by the amount of precipitation, and these results are consistent with those of previous studies^34^. In addition, strong yearly fluctuations in the insect communities were consistent with changes in the forb biomass, with gradual increases in insect species richness and abundance observed from 2007 to 2009 (Fig. 4). Interannual changes of insect species and abundance might be attributable to water availability^35^,^37^. Higher annual rainfall in 2008 may have provided better environmental condition for insect oviposition^38^, and the abundant individual of spring rainfall (April to June) in 2009 may have favored quick eggs hatching. Furthermore, a more productive plant community because of higher rainfall in 2008 may have provided more food resources for insects in August, which is a critical period of propagation^39^. These above causes likely increased the insect species richness and abundance in 2009, including that of herbivorous and predatory insects.

Our findings that warming had positive effects on the biomass of the entire plant community in the meadow steppe are consistent with the results observed in temperate grassland^16^, tallgrass pririe^36^, and Arctic tundra^40^. In the experimental area, grasses dominate the plant community, and *L. chinensis* is a dominant perennial rhizome grass species. The grass is the first plants to germinate from roots each year in these areas, and the results from our previous study showed that a higher accumulation of annual plant community biomass may have led to more plant litters^41^, which may have suppressed the germination and regrowth of *L. chinensis* (J. Liu *et al.* unpublished)^42^. Thus, the density of *L. chinensis* was reduced in the warmed plots (Fig. 3B), consequently leading to lower grass biomass (Fig. 2B). A decline in the dominance of *L. chinensis* could have allowed other annual forbs to rapidly colonize the plant community because of the weakened competitive ability of *L. chinensis*, thus driving the enhanced biomass and species richness of forbs (Fig. 2C,F). Annual repetitions of this process may have slightly increasedthe species richness of the plant community (Fig. 2A). Furthermore, these results indicate that warming shifted the grass-dominated plant community toward grass-forb mixed plant community due to decreased grass biomass and increased forb biomass, with slight changes occurring in the species richness of the grasses and forbs.

Shifts in plant community characteristics in the warmed plots had important consequences for higher trophic levels. According to bottom-up effects, slight increase in species richness and significant increase in the biomass of plant communities enhance insect diversity and abundance^25^,^26^. However, our results showed that insect species richness and abundance significantly decreased in the warmed plots (Fig. 4A,D). Our observations are consistent with the results observed in Arctic sites^11^, grasslands^42^, and other ecosystems^18^, and are inconsistent with other cases^19^. Some studies have shown that insect community changes may occur through mismatched phenology or altered distributions of insects and host plants^10^,^43^,^44^. Our empirical evidence suggests that other vegetation attributes explain the responses of insect species richness and abundance to climate warming.

The reduced in insect abundance observed in this study, including the abundance of the entire insect community and herbivorous insects under elevated temperature are mainly ascribed to changes in certain dominate insect species that are closely associated with grasses (Fig. 6). Thus, the overall insect abundance was affected by temperature because several dominant species presented an altered abundance in the warmer environment. In our experimental sites, the ratios of *E. unicolor* and Cicadellidae sp. were larger in the entire insect community (see the Materials and Methods section). The two dominant species are herbivorous insects that showed a preference for feeding on grasses^45^,^46^. Experimentation showed that climate warming reduced the biomass of grasses (11.5%), especially *L. chinensis* (Figs 2E,3B), the main plant resources for *E. unicolor* and Cicadellidae sp. A decrease in the quantity of food resources for herbivorous insects inevitably limits the growth and fecundity of insects^39^. Therefore, the negative effects on grass biomass induced by warming might have further harmed herbivorous insects because the abundance of *E. unicolor* and Cicadellidae sp. is positively related to grass biomass (Fig. 6C,D). On the other hand, increases in forb biomass, especially *Artemisia* forbs, could suppress the abundance of *E. unicolor* and Cicadellidae sp. the two most dominant herbivorous insects. Generally, the presence of *Artemisia* plants could play an important role in discoouraging herbivory^46^, because these plants produce secondary metabolites, such as flavonoid compounds, which reduced the food consumption by herbivorous insects, and strongly affect herbivore performance^47^,^48^. The negative relationships between the abundance of *E. unicolor* and Cicadellidae sp. and *Artemisia* biomass within all of the plots in this study further support our assertions (Fig. 6E,F), although the abundance of the two insects were not related to the species richness of *Artemisia* (data not shown). Thus, the abundance of *E. unicolor* and Cicadellidae sp. decreased owing to lower food resources and non-host plant defense, which resulted in a decline in total insect abundance.

For the entire insect community, herbivores and predators were the two dominant trophic levels (see the Materials and Methods section); thus, the responses in one trophic level to climate warming might affect other trophic levels via trophic cascading due to resource availability^25^. As shown above, experimental warming significantly decreased the abundance of herbivorous insects (Fig. 4E) and reduced the predator species richness because of trophic cascading effects (Fig. 4C) Furthermore, our results also showed that predators were positively associated with herbivores (Fig. 5B,C), indicating the potentially substantive feeding relationships between herbivores and predators. In the context of global warming, ectotherms are more sensitive to climate change^49^, and this sensitivity increas significantly with increasing trophic levels. Predatory insects may be generally more active, and have intrinsically higher metabolic rates, thus leading to rapid responses of predator to changes in the environments^50^. Conceivably, elevated temperature could also directly affect predators and produce a decline in predator species richness. Although evidence of the direct effects of temperature on predators was not observed in the study, it is likely that the reduced herbivore abundance partly contributed to the decreased predator species richness, because the quantity of food resource for predators are lower in the warmed plots. However, the warming treatments did not have significant effects on herbivore species richness in the study (Fig. 4B). Therefore, the reduction in predator species richness largely contributed to the decrease in total insect richness (Fig. 4).

We recognize that some caution must be taken with respect to interpreting our data because for highly mobile insects (such as species of grasshoppers that actively locate their hosts), their movement between plots is possible, thus influencing insect community at the time of our sampling. Our field observations indicated that insects can be immoblie under undisturbed conditions, the plots are separated by 2.5 m between plots as a buffer to allow the free movement of insects; therefore, the insects colonized the plots by naturally selecting hosts and habitat. We cannot exclude the possibility that several insect species moved between plots. However, we feel that our data could support the conclusion that the warming treatment had negative effects on the insect community over the three experimental years. Another concern is that this strong outcome is a potential artifact because of the small plot size, which may have prevented the observance of reliable effects on the insect community. We feel, however, that this observed outcome provides a reasonable approximation of field conditions for several reasons. First, insect species in the plots in the study showed little variation compared with other cases that included open field plots ranging from 25 m × 25 m to 3 m × 3 m^28^,^37^. Second, to ensure that the samplings were representative, the frequency of the samplings were increased (see methods and Zhu *et al.*^37^).

In addition, our results showed that the insect species richness and abundance were positively linked to the plant species richness under control conditions (Fig. 7A). Furthermore, the magnitude of these associations was significantly enhanced under the warmed conditions (Fig. 7B), suggesting that the dependence of insect diversity on plant diversity was significantly enhanced by elevated air temperature in the grassland ecosystem. To our knowledge, this study of community-level responses provides novel insights into the effects of climate change on the strength of plant-insect interactions. The strength of trophic interactions is one of the most important metrics of community dynamic, and it is strongly affected by climate warming^51^,^52^. Previous studies have shown that warmer temperatures can alter the phenology and distribution of individual species, consequently influencing the strength of plant-insect interactions^10^,^11^,^53^,^54^; but see the researches of de Sasii *et al.* and Ovaskainen *et al.*^14^,^55^. Although this experiment was not designed to directly manipulate plant diversity to examine its relationships with insect diversity, the study reveals that warming likely affects the strength of the plant-herbivore interactions by changing the interrelationship of their diversity. Our study identifies a need for future investigations to assess the strengthening effects of manipulated plant diversity on insect diversity under changing environmental conditions, which will improve our understanding the responses of multitrophic level interactions to climate change.

## Conclusions

Trophic interactions are widespread ecological phenomena in ecosystems, and they have been affected by climate change^9^,^56^. Herein, we provide evidence consistent with this hypothesis, and our study showed that insects experienced dramatic reductions in species richness and abundance under climate warming, which was partly because of their responses to the changed plant community induced by elevated air temperature. These results highlight the importance of empirical studies at the community level rather than at the species level, since individual species can present idiosyncratic responses that do not reflect average community-wide responses. Interestingly, our results showed that experimental warming enhanced the dependence of insect diversity on plant diversity, which implies that altered plant diversity under changing environmental conditions, could initiate bottom-up cascading effects to higher trophic levels. Studies of a single global driver would not have generated an adequate understanding of the community responses observed here, as multiple global change drivers (temperature, precipitation, and nitrogen) occur simultaneously, and may have synergistic effects in the real world. Only by scaling up our understanding of changes from the species to the community level under multiple global change drivers can we fully understand how current and future environmental changes are likely to affect biodiversity, community stability, and ecosystem functioning.

## Methods

### Study site

The experimental site is located at the Grassland Ecological Research Station of Northeast Normal University, Jilin Province, People’s Republic of China (44°45′N, 123°45′E). The site is in meadow steppe region where the mean annual temperature and precipitation in recent decades have range from 4.6 to 6.4 °C, and 280 to 400 mm, respectively. The monthly mean temperature ranges from -16 °C in January to 25 °C in July, and 90% of the total precipitation is distributed from April to October. The annual potential evaporation is approximately three times as high as the mean annual precipitation. The main vegetation type is meadow steppe predominated by *L. chinensis*^57^. Other species include perennial and annual grasses, such as *Phragmites australis, Calamagrostis epigejos*, and *Chloris virgata*; forbs, such as *Kalimeris integrifolia, Potentilla flagellaris*, and *Carex duriuscula*; and legumes, such as *Lespedeza davurica* and *Midicago ruthenica*. The soils are mixed saline and alkaline (pH 8.5-10.0).

### Experimental design and treatments

This experiment was set up in April 2006 and used a complete randomized block design at this site with homogeneous vegetation and flat topography^58^. Detailed information on plant community composition is described in Supplementary Table S1. Because the great difficulty of manipulating air temperature over larger area, six blocks were set up with an area of 8 × 4 m for each, with a pair of 4 × 3 m plots in each block, and the experimental area was similar to that in other studies^18^,^19^. The distance between neighboring plots was approximately 2.5 m. The paired plots in each block were randomly assigned to two temperature treatments: control and warming. The warming treatments were achieved by suspending 160 × 15 cm MSR-2420 infrared radiators (Kalglo Electronics Inc., Bethlehem, PA, USA) 2.25 m above the surface of all of the warming plots beginning in May 2006 (heaters were turned off over the winter from November 16 to March 15). Previous testing has shown that the infrared radiators do not generate visible light that can influence photosynthesis^59^. The effects of the infrared radiators on the soil temperature were spatially uniform within the warmed plots^60^. Experimentation determined that, at the experimental height, one heater with a radiation output of 1700 watts m^−2^ would warm the soil surface approximately 1.7 °C ( ± 0.1). Dummy heaters of the same shape, size and installation were placed in the control plots to artificially mimick the shading effect.

### Meteorological and soil temperature measurements

Meteorological measurements were performed beginning in 2007. The precipitation was measured with a tipping-bucket rain gauge (TE525MM, Texas Electronics, Dallas, TX, USA), and the air temperature was measured with a temperature probe (HMP45C, Vaisala Inc., Helsinki, Finland). The precipitation and temperature were recorded and averaged or summed over a 30 min interval by separate data loggers (CR3000, Campbell Scientific, Inc., Logan, UT, USA). The soil temperature was measured by using an ECH_2_O dielectric aquameter (Em50, Decagon Inc., USA), which automatically measured the soil temperature at a depth of 10 cm. These measurements were performed at 8:00-9:00 AM in late May, mid-June, mid-July, early August, mid-September and mid-October in 2007, 2008 and 2009. Five measurements were taken, and the average of the five was stored as the mean value per plot. Changes in the air and the soil temperatures, and precipitation measured in the experimental periods are provided in Supplementary Table S2, Figure S1,S2.

### Plant sampling

Plant measurements, included the plant species richness, individual number and individual plant species height, and they were performed four times at from June to September each year (from 2007 to 2009). Because this experiment was designed as a long-term manipulative experiment, all of the plant samplings were performed non-destructively. Plant species richness, vegetation height, and individual plant species number were recorded within each of the five randomly located 0.25 × 0.25 m quadrats in each plot. The species richness was considered the number of each plant species in all quadrat in each plot across all four samplings in every given year. The height of each plant species within a quadrat was calculated as the average of at least five random measurements of the species’ natural height. The AGB was obtained at peak standing biomass (mid-August in each year) and calculated using the plant height-biomass equation (see Supplementary Table S3 for the details of the plant AGB calculation, and see Zhu *et al.*^37^). To determine the responses of plant community composition to warming, the plants were divided into two functional groups: grasses and forbs. Because the legume species richness was only 1, and showed lower ratio of biomass over the three experimental years (<0.4%), these species were omitted in the analysis described below for the plant community. The species richness and biomass were calculated in each given year respectively.

### Insect sampling and identification

We followed the standard sweep net survey method (a light muslin net 35 cm in diameter) to estimate the insect species richness and abundance^24^,^37^. While sweep netting does not sample all of the insects in the community, community measurements obtained from sweep netting have been shown to be highly correlated with the insect parameters obtained by other methods in grasslands^23^, particularly suction sampling at this experimental site as showed by H. Zhu *et al.*(unpublished data). Insects were measured monthly between June and September from 2007 to 2009. Before the sampling, we ensured that the insects from the fields surrounding the sites naturally colonized the plants in each experimental plot (H. Zhu, personal observation). Insect specimens were collected under favorable monitoring conditions (sunny days with minimal cloud cover and calm or no wind) from 9:00 AM to 3:00 PM when the insects were most active. Each sample consisted of six beats with nets in each plot to ensure that the samples were representative. Six samplings were conducted in every plot, and these results were averaged over a 5-day interval in each month. To minimize the potential effects of insect immigration and migration on the data, 12 plots were swept simultaneously on each given sampling date. The contents of the sweep nets were preserved in bottles containing enough ethyl acetate to kill the insects. All of the individuals were identified to the species level. Specimens that could not be identified to species level were assigned to the lowest identifiable taxonomic category. The insects were assigned into four trophic categories: herbivores, predators, detritivores, and parasitoids; the two later guilds were not analyzed due to their lower abundance (1.8%) relative to herbivores and predators (98.2%). In our study, pre-mature insects were excluded for the species richness and abundance analyses. The insect species richness and abundance (including herbivores and predators) were recorded as the cumulative number of insect species and the accumulative abundance throughout the sampling periods in a given year. To account for the effects of abundance on the species richness, we used rarefaction^61^ to estimate the ‘expected species richness’ (i.e., rarefied richness, hereafter richness) in each plot. *E. unicolor* and Cicadellidae sp. were identified as the two dominant species in sampled insect community because of their higher abundance (*E. unicolor* 16.9%; Cicadellidae sp. 39.4%). The list of all insects that were collected from 2007 to 2009 is presented in Table S4.

### Data analyses

All of the data were tested for normality proit to performing the analyses. Three-way analysis of variance (ANOVAs) was performed to test the main and interactive effects of the block, year, and warming on the vegetation and insect variables. A general linear model (GLM) with Tukey’s post hoc test was used to examine significant difference in the mean values of the treatments. The effects of the blocks were tested together with the treatments in the above analyses; however, they were not discussed in this study due to the lack of significant difference. Independent-sample *t* tests were used to analyze the difference in vegetation and insect variables between the control and warmed treatments in each year. Before conducting the below analyses, Ln transformation was performed for the abundance of total insects, *E. unicolor*, and Cicadellidae sp., and the grass and *Artemisia* biomass, and the insect species richness were calculated. Linear regressions analyses were used to determine the effects of plant biomass (grasses and *Artemisia*) on insect abundance (*E. unicolor* and Cicadellidae sp.), the effects of herbivores on predators, and the effects of plant species richness on insect richness and abundance in both the warmed and control plots. An analysis of covariance (ANCOVA) was used to assess the treatment effects on insect variables, with plant species richness as a fixed factor, warming as the covariate, year as random factor, and species richness/abundance of all insects as dependent variables. Significance was set at *P* ≤ 0.05. All of the statistical tests were performed using SAS 9.13 statistical package (SAS Institute Inc., Cary, NC, USA).

## Additional Information

**How to cite this article**: Zhu, H. *et al.* Responses of community-level plant-insect interactions to climate warming in a meadow steppe. *Sci. Rep.* **5**, 18654; doi: 10.1038/srep18654 (2015).

## Supplementary Material

Supplementary Information

## Figures and Tables

**Figure 1:**
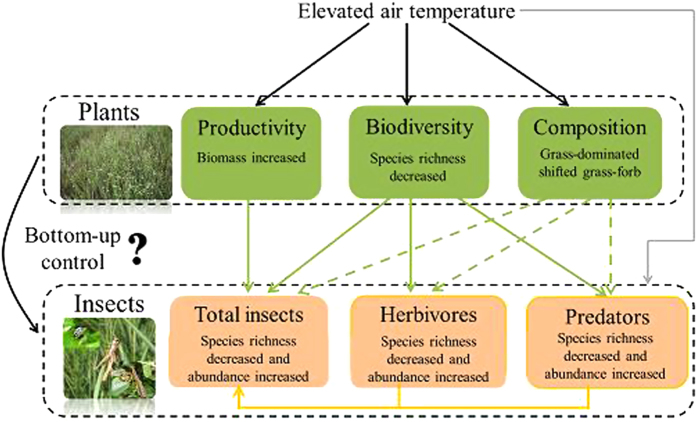
Potential mechanism underlying the effects of elevated temperature on plant-insect interactions. Dashed arrows represent the uncertain effects of warming, and solid arrows represent the mechanism examined in previous studies. Warming significantly affect productivity, species richness, and plant community composition (black lines). The altered plant community (increased plant biomass, decreased species richness, and changed plant community composition) cause cascade effects on insect community (green lines), including a decreased insect species richness and increased insect abundance. Changes in the herbivores and predators largely contributed to alterations in the entire insect community (orange-yellow lines). Thus, warming may have potential effects on bottom-up control of plant community on insect community. Gray lines represent the direct effects of warming on insects; however the effects were not examined in this study.

**Table 1:** Results of three-way ANOVA for the effects of block*, year, and warming and their interactions on the measured variables of vegetation and insects.

Variables	Year		Warming		Year × Warming	
	F	*P*	F	*P*	F	*P*
Total plant biomass	5.353	0.01	30.623	<0.0001	0.516	0.602
Grass biomass	111.61	<0.0001	14.611	0.001	1.352	0.274
Forb biomass	37.512	<0.0001	61.199	<0.0001	1.471	0.246
*Artemisia* biomass	60.058	<0.0001	41.549	<0.0001	1.885	0.169
Plant species richness	1.348	0.275	3.898	0.058	0.064	0.938
Grass species richness	2.261	0.122	2.13	0.155	0.174	0.841
Forb species richness	1.437	0.254	3.991	0.055	0.275	0.762
*Leymus chinensis* density	103.825	<0.0001	23.335	<0.0001	0.046	0.956
Insect species richness	35.992	<0.0001	19.967	<0.0001	0.393	0.679
Insect abundance	108.529	<0.0001	41.538	<0.0001	7.255	0.003
Herbivore species richness	30.445	<0.0001	3.341	0.078	0.591	0.56
Herbivore abundance	107.076	<0.0001	38.945	<0.0001	8.547	0.001
Predator species richness	12.93	<0.0001	27.552	<0.0001	0.482	0.622
Predator abundance	7.541	0.002	5.418	0.027	0.253	0.778
*Euchorthippus unicolor* abundance	1.685	0.203	22.784	<0.0001	0.205	0.816
Cicadellidae sp. abundance	26.907	<0.0001	32.26	<0.0001	3.156	0.057

*indicated the effects of blocks on the measured variables, the results are not shown in Table1due to insignificant effects.
