# Supplementary material for: Responses of community-level plant-insect interactions to climate warming in a meadow steppe
Source: Sci Rep. 2015 Dec 21;5:18654. doi: 10.1038/srep18654 (PMC4685464; doi:10.1038/srep18654)
Supplement: Supplementary Information [file srep18654-s1.pdf]

Responses of community-level plant-insect interactions to climate warming in a meadow steppe

Hui Zhu[1,2,3#], Xuehui Zou[1#], Deli Wang[1*], Shiqiang Wan[4], Ling Wang[1], and Jixun Guo[1]

Supplementary Information:

Table S1 The descriptions of plant community composition in experimental area in 2006.

Plant species	Block 1		Block 2		Block 3		Block 4		Block 5		Block 6	
	Control	Warming	Control	Warming	Control	Warming	Control	Warming	Control	Warming	Control	Warming
Leymus chinensis	√	√	√	√	√	√	√	√	√	√	√	√
Thalictrum simplex	√				√				√	√		√
Phragmites australis	√	√	√	√	√	√	√	√	√	√	√	√
Kalimeris integrifolia	√	√	√	√	√	√	√	√	√	√	√	√
Carex duriuscula	√	√	√	√	√	√	√	√	√	√	√	√
Artemisia mongolica	√	√	√	√	√	√	√	√	√	√	√	√
Artemisia anethifolia					√	√			√			
Potentilla flagellaris	√	√	√	√	√		√	√	√	√	√	√
Polygonum sibiricum		√	√			√	√			√		
Inula japonica			√	√			√	√			√	

Table S2 Results of three-way ANOVA for the effects of year (Y) and warming (W) and their interactions on soil temperature

Source of Variation	d.f.	F	*P*
Block	5,27	1.257	0.329
Y	2,27	38.27	< 0.0001
W	1,27	75.366	< 0.0001
Y × W	2,27	1.684	0.207

Table S3 The regression equations of height and biomass of each plant species in August each year.

Plant species	2007	2008	2009
Leymus chinensis	$y=0.002x^{1.996}$	$y=0.0003x^{1.863}$	$y=-4.4\times10^{-5}x^2+0.021x-0.368$
Phragmites australis	$y=0.001x^{1.859}$	$y=0.002x^{1.768}$	$y=0.405e^{0.026x}$
Kalimeris integrifolia	$y=9\times10^{-9}x^3+0.022x-0.268$	$y=6.4\times10^{-7}x^3+0.031x-1.263$	$y=2.2\times10^{-7}x^3+0.059x-1.416$
Puccinellia tenuiflora	$y=7.31\times10^{-5}x^{2.195}$	$y=5.84\times10^{-5}x^{2.432}$	$y=6.47\times10^{-5}x^{2.682}$
Artemisia scoparia	$y=0.017e^{0.114x}$	$y=0.019e^{0.105x}$	$y=0.002x^2-0.07x+0.967$
Artemisia mongolica	$y=0.132e^{0.046x}$	$y=5.64\times10^{-5}x^{2.639}$	$y=5.35\times10^{-5}x^{2.727}$
Inula japonica	$y=8.51\times10^{-5}x^3+0.003x^2+0.04x-0.124$		
Carex duriuscula	$y=0.007e^{0.096x}$	$y=0.009e^{0.084x}$	$y=0.146e^{0.032x}$
Polygonum sibiricum	$y=0.051e^{0.16x}$		$y=0.003x^{1.937}$
Thalictrum simplex	$y=9.9\times10^{-5}x^{2.654}$	$y=0.06x^{1.038}$	$y=0.01x^{1.244}$
Calamagrostis epigejos		$y=0.0002x^{2.015}$	

Calculation of height-biomass equation of plant species

The method of calculation of plant height-biomass equation was as follows: at least 100 individuals of each plant species that were present in the warmed and control plots were harvested by clipping plants shoot 1 cm above the soil surface in nearby warming treatments plots (ranging from 2-3 m to exclude the heterogeneity of soil). Each clipped individual was measured height, dried at 80°C for 48 h, and weighed. The regression equation of each plant species was calculated by height and biomass of individual using simple regressions. The statistical significance of linear or

curvilinear equation was the higher, and then the equation can act as plant height-biomass equation of this plant species. Detailed descriptions of biomass-height equations of all plant species in August from 2007 to 2009 are presented in Table S1.

Individual biomass of each plant species in experimental plots was calculated by height and regression equation of plant biomass-height of this plant species. Plant biomass in each quadrat was obtained through cumulative individual biomass of plant species, excluding for rare species (legumes). Aboveground plant biomass was calculated as per unit biomass that total biomass in five 0.25 × 0.25 m quadrats was transformed, including grasses, forbs, and total plant biomass.

Table S4 The list of all insects collected in three experimental years, and individual numbers of each species.

**Order**	**Family**	**Species**	**Number**
Orthoptera	Arcypteridae	*Euchorthippus unicolor*	1448
		Euchorthippus vittatus	129
	Oedipodidae	*Epacromius coerulipes*	122
		Epacromius tergestinus	100
		Oedaleus infernalis	13
	Acrididae	*Acrida cinerea*	9
	Pyrgomorphidae	*Atractomorpha sinensis*	87
	Tettigoniidae	*Gampsocleis ussuriensis*	22
Hemiptera	Pentatomidae	*Aelia fieberi*	109
		Euryderma gebleri	57
		Dolycoris baccarum	
	Lygaeidae	Lygaeidae sp.1	42
		Lygaeidae sp.2	120
		Lygaeidae sp.3	3
	Nabidae	Nabidae sp.1	1
		Nabis stenoferus	39
	Coreidae	*Corizu tetraspilus*	2
	Miridae	*Adelphocoris suturalis*	56
		graphosoma rubrolineata	150
		Adelphocoris fasciaticollis	3
		Miridae sp.1	1
Coleoptera	Curculionidae	Curculionidae sp.1	5
		Curculionidae sp.2	359
		Chlorophanus lineolus	4
	Coccihellidae	*Coccinula quatuordecimpustulata*	261
		Coccinella septempunctata	1
		Propyiea japonica	36
		Harmonia axyridis	48
		Hippodamia variegata	3
		Hyperaspis gyotokui	9
	Chrysomelidae	*Cryptocephalinae* sp.1	47
		Chrysomelidae sp.1	1
		Monolepta hieroglyphica	53
		Basilepta fulvipes	247
		Labidostomis chinensis	6
	Meloidae	*Epicauta chinensis*	50
		Mylabris calida	9
	Elateridae	Elateridae sp.1	4
		Coleoptera sp.1	62

Diptera	Sarcophagidae	Sarcophagidae sp.1	116
	Trypetidae	Trypetidae sp.1	32
		Trypetidae sp.2	30
		Trypetidae sp.3	44
	Calliphoridae	Calliphoridae sp.1	20
		Calliphoridae sp.2	161
	Chloropidae	Chloropidae sp.1	310
		Chloropidae sp.2	192
	Anthomyiidae	Anthomyiidae sp.1	45
	Tabanidae	Tabanidae sp.1	7
	Empididae	Empididae sp.1	9
	Bombyliidae	Bombyliidae sp.1	29
	Asilidae	Asilidae sp.1	8
		Asilidae sp.2	5
		Asilidae sp.3	5
	Tachinidae	Tachinidae sp.1	18
		Tachinidae sp.1	8
		Diptera sp.1	28
Hymenoptera	Ichneumqnidae	*Ophion luteus*	4
		Ichneumqnidae sp.1	2
		Ichneumqnidae sp.2	12
		Ichneumqnidae sp.3	8
		Ichneumqnidae sp.4	24
		Ichneumqnidae sp.5	10
	Apidae	Apidae sp.1	3
		Apidae sp.2	7
		Apidae sp.3	2
	Sphecidae	Sphecidae sp.1	8
	Pompilidae	Pompilidae sp.1	3
	Tenthredinidae	Tenthredinidae sp.1	26
	Megachilidae	Megachilidae sp.1	11
	Eumenidae	Eumenidae sp.1	1
		Eumenidae sp.2	2
		Eumenidae sp.3	1
Homoptera	Cicadellidae	Cicadellidae sp.1	3
		Cicadellidae sp.2	8
		Cicadellidae sp.3	3382
		Cicadella viridis	10
Lepidoptera	Noctuidae	Noctuidae sp.1	65
		Noctuidae sp.2	15
		Noctuidae sp.3	70
		Noctuidae sp.4	2
		Noctuidae sp.5	2
	Lycaenidae	Lycaenidae sp.1	1

	Geometridae	Geometridae sp.1	10
		Geometridae sp.2	6
Neuroptera	Chrysopidae	*Sympetrum Croceolum*	45
Mantedea	Mantidae	*Mantis religiosa*	23

Figure S1

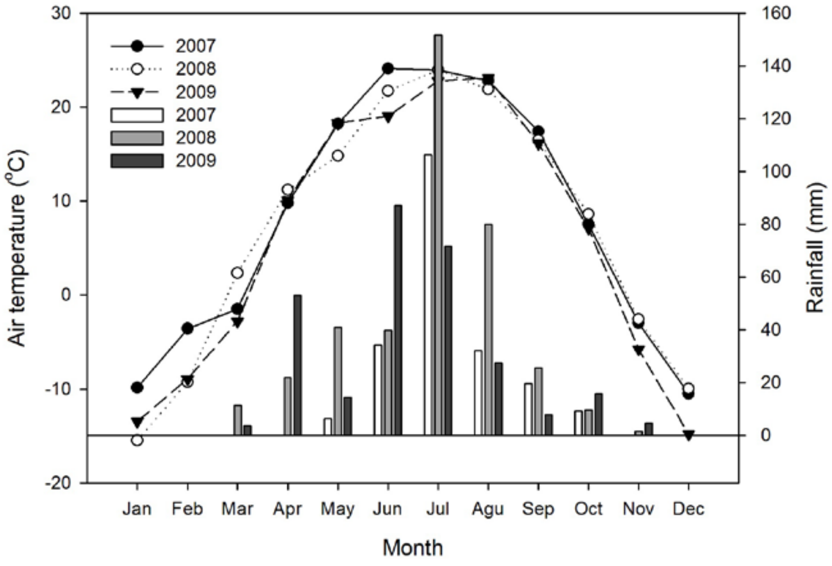

Figure S1 Monthly air temperature (lines) and rainfall (columns) from 2007 to 2009.

Figure S2

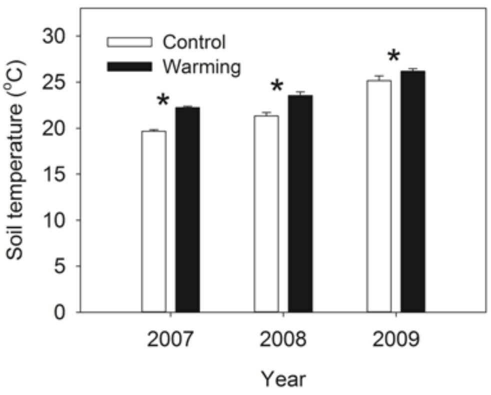

Figure S2 Soil temperature (0-10 cm) in control and warmed treatments in 2007, 2008, and 2009. Asterisk indicates a significant difference between warmed and unwarmed treatments in each given year, and * $P < 0.05$.
